# Efficacy of low-dose risperidone in combination with sertraline in first-episode drug-naïve patients with schizophrenia: a randomized controlled open-label study

**DOI:** 10.1186/s12967-023-04272-7

**Published:** 2023-07-04

**Authors:** Xiaoe Lang, Mei Xue, Xiaocui Zang, Fengchun Wu, Meihong Xiu, Xiangyang Zhang

**Affiliations:** 1 Department of Psychiatry, First Hospital of Shanxi Medical University, Taiyuan, China; 2 Qingdao Mental Health Center, Qingdao, China; 3 Peking University HuiLongGuan Clinical Medical School, Beijing HuiLongGuan Hospital, Beijing, China; 4 Department of Psychiatry, The Affiliated Brain Hospital of Guangzhou Medical University, Guangzhou, China; 5 Guangdong Engineering Technology Research Center for Translational Medicine of Mental Disorders, Guangzhou, China; 6 CAS Key Laboratory of Mental Health, Institute of Psychology, Beijing, China; 7 Department of Psychology, University of Chinese Academy of Sciences, Beijing, China

**Keywords:** Schizophrenia, Risperidone, First-episode, Sertraline, Efficacy

## Abstract

**Objective:**

Despite advances in pharmacology, the treatment of schizophrenia (SZ) remains a challenge due to relapse after antipsychotic discontinuation and multiple adverse effects of antipsychotics. We hypothesized that a low dose of risperidone in combination with sertraline would reduce serious adverse effects without decreasing treatment response. This study aimed to examine the efficacy, safety, and tolerability of low-dose risperidone combined with sertraline to reduce risperidone dose and serious adverse effects in first-episode and medication-naive (FEMN) SZ patients.

**Methods:**

A total of 230 patients with FEMN SZ were randomly assigned to receive low-dose risperidone in combination with sertraline (RS group) or regular-dose risperidone (control group). The Positive and Negative Syndrome Scale (PANSS), Hamilton Depression Rating Scale (HAMD), and Personal and Social Performance Scale (PSP) were assessed at baseline and the end of the first, second, third, and sixth months. In addition, serum prolactin levels and extrapyramidal symptoms were measured at baseline and follow-up.

**Results:**

Repeated measures ANCOVA showed significant interaction effects of treatment by time on psychotic symptoms, as well as HAMD, PSP scores, prolactin levels, and extrapyramidal symptoms (all p < 0.05). Compared with the control group, the RS group had greater decreases in PANSS total score and its subscores and HAMD score (all p < 0.01) and a greater increase in PSP total score (p < 0.01). Notably, side effects were lower in the RS group relative to the control group. Improvements in HAMD and PANSS total scores, changes in prolactin levels and gender predicted improvements in PSP from baseline to month 6.

**Conclusions:**

Our study suggests that low-dose risperidone in combination with sertraline was more effective for psychotic symptoms and psychosocial functioning, with significantly fewer adverse effects in patients with FEMN SZ.

*Trial registration number*: ClinicalTrials.gov, NCT04076371

**Supplementary Information:**

The online version contains supplementary material available at 10.1186/s12967-023-04272-7.

## Introduction

Schizophrenia (SZ) is a severe, chronic mental disorder with a significant decline in psychosocial functioning [1]. Patients with SZ usually present with psychotic symptoms and chronic decline in cognitive functioning as well as emotional disturbances. The etiology of SZ is unknown, with a high rate of relapse and unresponsiveness to antipsychotics, resulting in family burden and family distress [2]. Currently, atypical antipsychotics are commonly prescribed to alleviate psychotic symptoms in SZ [3]. However, the efficacy of antipsychotic medications does not achieve satisfactory clinical response, and it is estimated that approximately 30% of patients with SZ exhibit non-response to antipsychotic medications [4, 5]. In particular, patients with SZ usually present with negative symptoms, which remains a key treatment challenge and a major target for novel treatment strategies [6].

Risperidone, one of the atypical antipsychotics used to treat SZ, is recommended as a first-line agent and is an effective treatment strategy to alleviate acute and chronic psychosis in patients with SZ at admission [7–9]. Previous large studies of relapse in patients treated with risperidone or haloperidol found a significantly lower relapse rate in the risperidone group [10]. In another meta-analysis, risperidone, amisulpride, and olanzapine were shown to be more effective than other antipsychotics in SZ patients [11]. Atypical antipsychotics cause different degrees and types of side effects due to different affinities for different receptors, and recent studies comparing the adverse effects of different antipsychotics on SZ have confirmed that antipsychotic-induced side effects differ significantly among patients with first-episode psychosis [12–14]. Risperidone ranks high in the list of antipsychotics associated with significantly elevated prolactin levels [11]. In addition, risperidone is associated with extrapyramidal symptoms and a high rate of antiparkinson medication use in patients with SZ, although a lower rate compared to haloperidol [15, 16]. Based on the CATIE study, it was reported that risperidone may cause akathisia in 7% of SZ patients [17].

Risperidone can drastically improve clinical symptoms in patients with SZ, but its role is limited due to the high incidence of hyperprolactinemia and extrapyramidal adverse effects. Exploring new combinations of antipsychotics to improve efficacy and reduce side effects in the treatment of SZ is an interesting topic. To reduce the adverse effects of risperidone, low doses of risperidone can be helpful for patients with SZ. However, there is a need to investigate a new clinical treatment option with fewer adverse effects and better efficacy than standard doses of risperidone. Given the negative symptoms and impairment of social functioning in SZ patients, we chose to use a selective 5-hydroxytryptamine reuptake inhibitor (SSRI) in combination with low-dose risperidone to alleviate symptoms in SZ patients. SSRIs have selective and potent inhibitory effects on presynaptic 5-HT reuptake and increase the 5-HT concentration in the synaptic gap, thereby promoting the action of the 5-hydroxytryptamine system. Importantly, sertraline has been reported to be the most potent SSRI in increasing extracellular DA concentrations in the striatum, and thus improving negative symptoms [18, 19]. Therefore, the combination with risperidone has the potential to alleviate negative symptoms and social functioning in SZ patients [20]. Sertraline is currently approved by the US Food and Drug Administration for the treatment of obsessive–compulsive disorder, self-injury, aggression, and depression in children aged 6 to 17 years [21]. Furthermore, it has a low activating effect compared to other psychotropic drugs and minimal metabolic interactions with other drugs compared to other SSRIs.

In the present study, we hypothesized that a low daily dose of risperidone combined with sertraline would be superior to standard doses of risperidone (4–6 mg/day) in improving psychosocial functioning and reducing adverse effects in FEMN SZ patients. Although risperidone can be utilized up to 16 mg/day, the purpose of this study was to investigate whether low-dose combination therapy would improve negative symptoms and social functioning, and reduce side effects more than the recommended dose of risperidone. Therefore, the manufacture’s recommended dose of risperidone (4–6 mg/day) was chosen for patients in the control group. In this 24 week open-label study, we investigated the following questions in patients with FEMN SZ: (1) to compare the improvement in psychotic symptoms in the low-dose risperidone combined with sertraline group (RS) and the risperidone monotherapy group (control group); (2) to compare the improvement in psychosocial functioning in the RS and control groups; (3) to compare the two groups in terms of improvements in depression and anxiety symptoms; (4) to compare the adverse effects of the two groups; and (5) is improvement in psychosocial functioning associated with a reduction in psychotic symptoms or adverse effects?

## Methods

### Subjects

This study was conducted at First Hospital of Shanxi Medical University. The study procedures and protocol were approved by the Ethics Committee of First Hospital of Shanxi Medical University and all participants or their guardians signed written informed consent.

A total of 263 patients from First Hospital of Shanxi Medical University were screened. Eligible adult patients (18–45 years) with SZ diagnosed according to the Diagnostic and Statistical Manual of Mental Disorders, fourth edition (DSM-IV) and Structured Clinical Interview for DSM Disorders (SCID) were recruited. All patients were first-episode medication-naïve patients (FEMN) with SZ. The complete eligibility criteria were shown as follows: (1) male or female outpatients; (2) 18–45 years of age; (3) disease duration of SZ not exceeding 60 months; (4) antipsychotic naïve; and (5) more than 9 years of education. Exclusion criteria were: (1) substance dependence except for nicotine; (2) major somatic comorbidities; and (3) abnormal values on routine biochemical tests.

Thirty-three patients were excluded and 230 patients were randomized to two treatment groups (115 in each group). The reasons for the exclusion included ineligible (n = 10), not meeting inclusion criteria (n = 9) and refusal to participate (n = 14).

### Study design

This was a 24 week, randomized, controlled open-label study. Patients were randomly assigned to receive low-dose risperidone (2–3.5 mg/day) and sertraline (50–100 mg/day) in the RS group (n = 109 patients) or normal-dose risperidone (4–6 mg/day) monotherapy in the control group (n = 89 patients) (Additional file 1: Fig. S1). First, a computer generated a sequence of random numbers. Then, an independent third party randomized patients to the RS and control groups based on their assigned computer-generated sequence of numbers. Interviewers remained blinded to treatment assignment throughout the trial. Patients were followed up for 24 weeks. The trial consisted of five visits, conducted at baseline, month 1, month 2, month 3, and month 6. Nurses ensured treatment adherence during the study period.

Recruited participants were allowed to take clonazepam (2–3 mg/d) during the early phase of treatment to treat sleep problems and relieve anxiety symptoms and acute agitation. Past medical history, ECG, and physical examination reports were obtained from all participants or their relatives.

### Treatment outcomes

The main outcome was clinical symptoms assessed by PANSS [22]. The endpoint was the changes from baseline PANSS scores to the value in the sixth month. Secondary efficacy outcomes were depressive symptoms as evaluated by the Hamilton Depression Rating Scale (HAMD) [23], psychosocial functioning as evaluated by the Personal and Social Performance Scale (PSP) [24], and CGI-S [25], and extrapyramidal symptoms (EPS) assessed by Extrapyramidal Symptom Rating Scale (ESRS) [26]. These outcomes were assessed at baseline, at the end of months 1, 2, 3, and 6.

In addition, blood prolactin levels were determined in the hospital laboratory using commercially available kits.

### Statistical analysis

Required sample sizes for adequate power were calculated using the G*Power program. Based on a one-sided α of 0.05, a sample size of 102 evaluable patients in each treatment group was needed to identify the effect size of 0.35 at 80% power. Assuming a dropout rate of 10%, a minimum sample of 204 evaluable patients (102 patients in each group) was needed.

Data missing for patients who dropped out were imputed via the last observation carried forward (LOCF) method. All analyses to evaluate between-group differences were conducted for an intent to treat (ITT) analysis set that included all the patients who received treatment. Demographic characteristics, baseline psychiatric symptoms, depressive symptoms, and psychosocial functioning between the RS and control groups were analyzed by one-way analysis of variance (ANOVA).

A repeated measures multivariate analysis of variance (RM-MANOVA) was used to compare the efficacy of the two treatment regimens. For dependent variables, prolactin levels and symptom scores measured at 5 time points were considered as repeated measures within effects, treatment groups (RS and control groups) were considered as between effects, and age, gender, age at onset, and education were covariates. In each RM-ANOVA model, the independent variables were outcome measures over time. If significant differences were observed in the interaction effect of the treatment group by time, an analysis of covariance (ANCOVA) with baseline value as covariates were conducted to examine differences in symptom scores assessed at different time points (months 1, 2, 3, and 6). In addition, ANCOVA was performed to compare the changes in symptom scores from baseline to follow-up.

Linear regression analysis was performed to identify variables associated with improvement in primary or secondary outcome measures. The following variables were potentially associated with the outcome measure and were therefore added to the regression model: baseline symptoms, gender, age, age at onset, duration of illness, and education.

Data were analyzed using PASW Statistics (SPSS 22.0, Inc., Chicago). Significance was set at p < 0.05 for all analyses. Given the multiple comparisons in the multivariate analysis, Bonferroni’s correction was used.

## Results

### Baseline characteristics

**Fig. 1:**
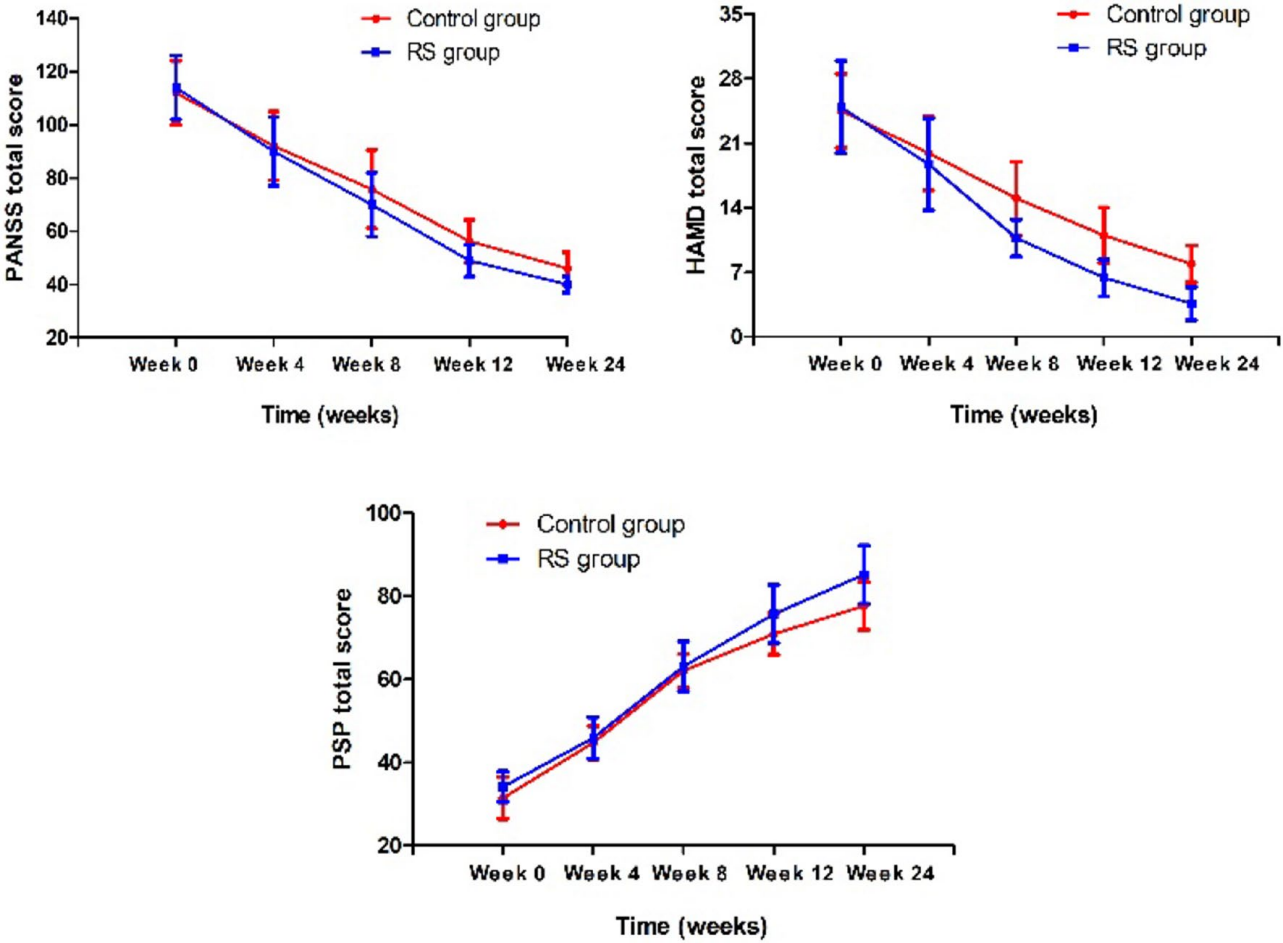
Combination therapy significantly improved clinical symptoms and psychosocial functioning relative to the control group (all p < 0.05)

Eligible patients were randomized to the RS group (n = 115) and the control group (n = 115). A total of 32 patients dropped out in the follow-up (n = 6 in the RS group and n = 26 in the control group). In the RS group, 9 patients discontinued for side effects caused by antipsychotics and 3 patients were lost to follow-up. In the control group, 21 patients discontinued for side effects caused by antipsychotics and 5 patient was lost to follow-up. The rate of dropout was higher in the control group (n = 26) than in the RS group (n = 6) (Fig. 1). A total of 230 patients were included in the efficacy ITT-population analysis.

At baseline, there were no significant differences between the RS and control groups in terms of age, gender, duration of disease, age at onset, and education (all p > 0.05). In addition, there were no significant differences in PANSS scores, CGI-S, PSP, and HAMD total scores between the two groups (all p > 0.05). In addition, there was also no difference in prolactin levels between the RS and control groups (p > 0.05).

At baseline, correlation analysis showed that serum prolactin levels were negatively correlated with the duration of disease in SZ patients (r = − 0.18, p = 0.008). PANSS total score was positively correlated with HAMD, and CGI-S scores (all p < 0.001) and negatively correlated with PSP total score (r = − 0.60, p < 0.001). In addition, PANSS total score was associated with age, gender, and duration of disease (all p < 0.05). Therefore, age, gender and age of onset were added as covariates in the following analysis (Table 1).

**Table 1:** Demographic characteristics and clinical data in combination therapy group (RS) and control group (mean ± SD)

Variable	Control group (n = 115)	RS group (n = 115)	F or *X*^2^ (p value)
Gender(male/female)	62/53	66/49	0.3 (0.60)
Age (years)	26.2 ± 6.6	26.1 ± 5.9	0.01 (0.92)
Education (years)	11.9 ± 2.3	12.4 ± 2.5	2.2 (0.14)
Age of onset (years)	24.0 ± 5.8	23.6 ± 5.2	0.2 (0.67)
Duration of illness (years)	2.2 ± 1.6	2.5 ± 1.8	1.8 (0.19)
Prolactin (ug/ml)	254.7 ± 54.9	259.1 ± 59.5	0.6 (0.44)
P	24.9 ± 6.2	23.9 ± 6.3	1.4 (0.24)
N	28.9 ± 6.1	30.2 ± 7.2	2.3 (0.14)
G	58.3 ± 8.8	59.9 ± 8.8	1.7 (0.19)
PANSS total score	112.2 ± 12.5	114.0 ± 13.4	1.2 (0.27)
HAMD total score	24.3 ± 4.8	24.8 ± 5.7	0.4 (0.51)
PSP total score	34.9 ± 5.2	34.2 ± 3.6	1.7 (0.20)
CGI-S total score	5.9 ± 0.7	5.8 ± 0.6	0.1 (0.77)

*SD* Standard Deviation, *P* positive subscore, *N* negative subscore, *G* general psychopathology subscore, *CGI-S* Clinical Global Impression-severity, *PSP* Personal and Social Performance Scale, *HAMD* Hamilton Depression Rating Scale

### Primary outcomes

RM-MANCOVA revealed a significant group-by-time interaction effect on PANSS scores, controlling for age, years of education, gender, and age at onset (Wilks’ lambda F = 55.4, p < 0.001) (Fig. 1). In univariate RM-ANCOVA analysis, significant group-by-time interaction effects for PANSS total score (F = 39.2, p < 0.001) and its 3 subscores (all p < 0.01) (Table 2) (Bonferroni Corrected p < 0.01).

**Table 2:** PANSS scores, HAMD score, CGI-S and PSP at baseline, week 4, week 8, week 12 and week 24 in first episode drug-naïve patients with schizophrenia treated with risperidone with sertraline (RS) group and risperidone monotherapy (control) group (mean ± SD)

	Baseline (n = 230)	Week 4 (n = 230)	Week 8 (n = 230)	Week 12 (n = 230)	Week 24 (n = 230)	Group (p value)^a^	Group × Time F(p value)^a^
PANSS positive subscore						5.1 (0.03)	8.2 (0.002)
Control group	24.9 ± 6.2	17.6 ± 5.1	14.8 ± 5.1	12.5 ± 5.1	11.1 ± 5.4		
RS group	23.9 ± 6.3	17.9 ± 5.1	13.4 ± 4.3	10.5 ± 3.9	9.1 ± 3.7		
PANSS negative subscore						25.0 (< 0.001)	78.0 (< 0.001)
Control group	28.9 ± 6.1	24.2 ± 7.1	21.5 ± 6.6	18.6 ± 6.0	15.8 ± 5.2		
RS group	30.2 ± 7.2	24.0 ± 7.2	19.5 ± 6.8	15.5 ± 4.8	11.0 ± 2.4		
PANSS general psychological subscore						9.6 (0.002)	24.2 (< 0.001)
Control group	58.3 ± 8.8	48.0 ± 9.4	40.9 ± 9.5	31.0 ± 9.1	27.6 ± 10.0		
RS group	59.9 ± 8.8	48.0 ± 8.9	37.5 ± 9.2	25.1 ± 5.8	22.6 ± 6.1		
PANSS total score						19.8 (0.001)	39.2 (< 0.001)
Control group	112.7 ± 12.8	92.0 ± 13.1	75.8 ± 13.7	56.3 ± 8.5	46.3 ± 6.2		
RS group	114.1 ± 13.6	90.4 ± 13.7	69.6 ± 14.0	49.2 ± 6.1	40.4 ± 3.1		
CGI-S score						30.9 (< 0.001)	42.2 (< 0.001)
Control group	5.9 ± 0.7	4.8 ± 0.4	4.0 ± 0.5	3.6 ± 0.7	3.2 ± 1.0		
RS group	5.8 ± 0.6	4.8 ± 0.6	3.9 ± 0.6	3.0 ± 0.7	2.2 ± 1.0		
HAMD total score						85.1 (< 0.001)	90.5 (< 0.001)
Control group	24.3 ± 4.8	19.0 ± 5.3	15.3 ± 4.6	12.5 ± 4.1	9.7 ± 4.6		
RS group	24.8 ± 5.7	18.5 ± 5.4	11.0 ± 3.2	6.9 ± 2.8	4.2 ± 3.3		
PSP total score						34.2 (< 0.001)	47.7 (< 0.001)
Control group	34.9 ± 5.2	44.6 ± 3.7	58.0 ± 8.9	64.8 ± 12.4	70.1 ± 15.2		
RS group	34.2 ± 3.6	45.9 ± 4.9	62.2 ± 7.3	74.1 ± 9.6	83.2 ± 11.3		

^a^Adjusted *F* value controlling for sex, age, and onset age

The ANCOVA analysis also showed that after 6 months of treatment, the RS group had greater improvements in the PANSS total score and its subscores after controlling for baseline values (all p < 0.01). After covarying for covariates, we found that the PANSS total scores were significantly lower in the RS group than in the control group (p < 0.01).

### Secondary outcomes

**Fig. 2:**
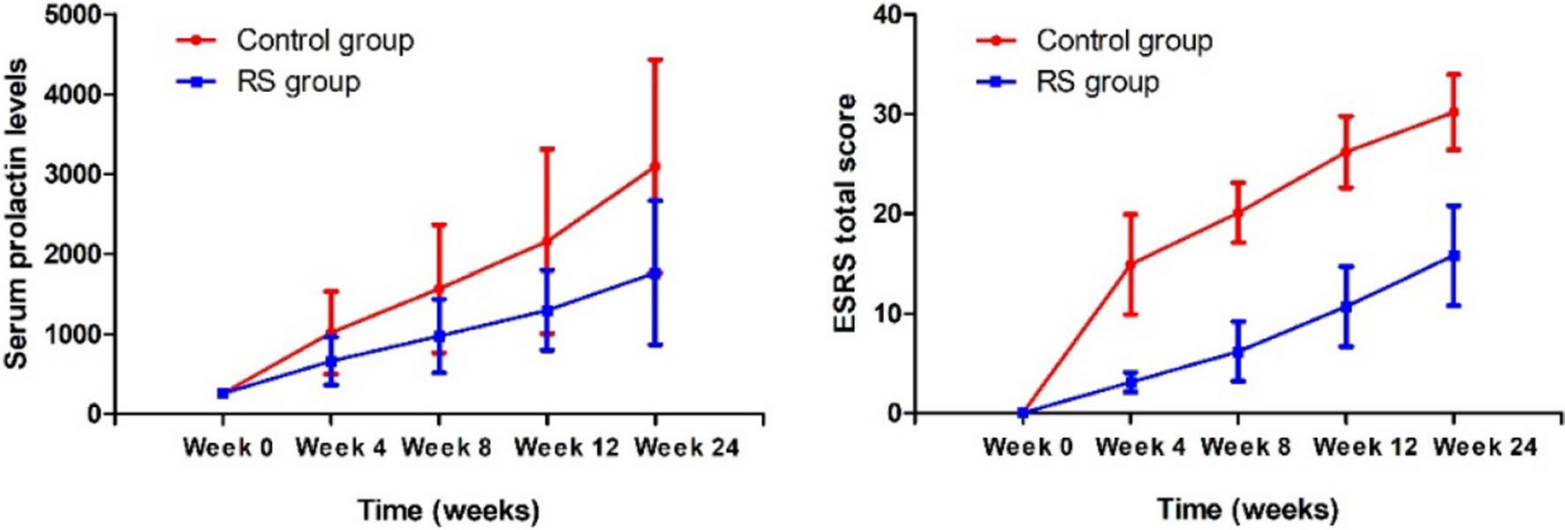
Combination therapy significantly reduced more adverse effects relative to the control group (all p < 0.05)

We found significant group-by-time interaction effects for CGI-S, PSP and HAMD scores, as well as prolactin levels and ESRS total score (all p < 0.05) (Tables 2, 3) (Fig. 2) (Bonferroni Corrected p < 0.01 for prolactin levels and Bonferroni Corrected p > 0.05 for ESRS total score). After controlling for the baseline values, there were significant differences between the RS and control groups in the changes in CGI-S, PSP, HAMD total scores, prolactin levels, and ESRS total score. At the 6 month follow-up, the RS group had significantly higher PSP total scores and lower HAMD total scores and prolactin levels than the control group (all p < 0.05).

**Table 3:** Interactive effects of group-by-time on EPS scores and prolactin levels (mean ± SD)

	Baseline (n = 230)	Week 4 (n = 230)	Week 8 (n = 230)	Week 12 (n = 230)	Week 24 (n = 230)	Group (p value)^a^	Group × Time F (p value)^a^
ESRS total score						< 0.001	4.2 (0.028)
Control group		12.2 ± 7.2	16.1 ± 8.1	20.8 ± 10.4	24.0 ± 12.1		
RS group		3.0 ± 1.7	5.9 ± 3.2	10.2 ± 4.7	15.1 ± 5.8		
Benzhexol						< 0.001	9.1 (< 0.001)
Control group		3.8 ± 1.3	4.5 ± 2.0	5.2 ± 2.2	5.5 ± 2.4		
RS group		1.4 ± 0.9	2.1 ± 1.3	2.9 ± 1.6	3.6 ± 1.9		
Prolactin levels						< 0.001	18.9 (< 0.001)
Control group	254.7 ± 55	846.3 ± 556	1272.0 ± 894	1728.6 ± 1289	2456.7 ± 1671		
RS group	259.1 ± 59	641.9 ± 310	939.2 ± 482	1244.7 ± 624	1688.1 ± 997		

^a^Adjusted *F* value controlling for sex, age, education years and onset age

However, there was a positive correlation between the decrease in CGI-S and the increase in PSP total score (r = 0.83, p < 0.001), and between the increase in PSP total score and the decrease in PANSS and HAMD total scores (both p < 0.01). Interestingly, we also found significant associations between an increase in prolactin levels and decreases in PANSS, HAMD, or an increase in total PSP scores (all p < 0.05). We added age, gender, duration of illness, baseline PSP and decreases in PANSS, HAMD, and CGI-S scores as covariates in our regression model and found that decrease in HAMD and PANSS total scores, change in prolactin levels and gender were predictors of improvement in PSP (*R*^2^ = 0.64).

In addition, patients in the RS group had fewer extrapyramidal symptoms and used less benzhexol compared to controls (Table 3).

## Discussion

The main findings of this study were that (1) after 6 months of treatment, low-dose risperidone in combination with sertraline was more efficacious than standard-dose risperidone alone in terms of psychotic symptoms and depressive symptoms; (2) the RS group was more efficacious than the control group in terms of improvement in psychosocial functioning; (3) the RS group had fewer adverse effects than the control group, including prolactin levels and EPS symptoms; and (4) improvement in psychosocial functioning was associated with changes in clinical symptoms and prolactin levels.

We found that low-dose risperidone combined with sertraline treatment for 24 weeks was more effective than risperidone monotherapy in alleviating negative symptoms, improving social functioning and reducing side effects in patients with FEMN SZ. That is, 24 weeks of low-dose risperidone combined with sertraline not only improved the efficacy in patients with FEMN SZ but also reduced adverse effects, such as elevated EPS and serum prolactin, thereby improving the safety of risperidone in FEMN patients. Furthermore, in line with other studies, the addition of sertraline was effective in improving depressive symptoms in SZ patients [27]. Therefore, this study provides a new combination antipsychotic regimen of low-dose risperidone plus sertraline for the early treatment of SZ in the clinical setting. Of particular note is that the use of combination therapy in FEMN SZ patients was not based on a certain threshold of depression symptoms. Although antidepressant treatments may be risky in this population, as previous studies have reported that antidepressant use is associated with an increased risk of suicide in some patients (suicidal ideation cannot be ignored as an adverse effect) [28, 29], in our study, no suicidal ideation was reported in FEMN SZ patients after 24 weeks of sertraline use. We speculate that this may be due to the lower than usual dose of sertraline taken by the patients in this study..

We hypothesize that the better efficacy of the combination of antipsychotics for SZ is due to the modulatory effect of sertraline on the 5-HT system. The effect of risperidone on the brain is mainly to reduce the activity of the dopaminergic pathway, thus reducing psychotic symptoms [30]. Negative symptoms and depressive symptoms have been reported to be associated with the dysfunction of DA and 5-HT neurons. However, the effect of risperidone on the 5-HT receptors in the brain was minimal. We found that the combination was efficacious in negative and depressive symptoms in SZ patients, which may be due to the selective inhibition of 5-HT reuptake by sertraline. Indeed, sertraline could improve the reduction of depressive symptoms by enhancing the activity of central dopaminergic neurons through the inhibition of DA transporters and reuptake [31, 32]. Moreover, the fact that low-dose risperidone combined with sertraline is more effective in reducing positive symptoms of SZ may also be due to the impact of sertraline. It may be that sertraline significantly reduced depression and negative symptoms by reducing positive symptoms of SZ. Risperidone, which targets DA activity, significantly improves positive symptoms closely related to DA dysfunction in the brain striatal, while increased 5-HT levels of sertraline may block DA activity and further improve positive symptoms of SZ [33, 34]. In addition, previous studies support that sertraline in combination with antipsychotics may reduce positive symptoms in SZ patients [35, 36]. Thus, the pharmacological effects of sertraline may explain the advantages of this promising combination treatment strategy for patients with SZ.

Notably, we found lower adverse effects in the RS group, including prolactin levels and EPS symptoms than with risperidone monotherapy. Evidence supports that high dose of atypical antipsychotics (e.g. olanzapine or risperidone) causes adverse effects in Parkinson’s disease at rates similar to those of low potency typical antipsychotics (chlorpromazine) [37]. Our findings provide further evidence that low-dose of antipsychotic combinations do not lead to an increased overall risk of antipsychotic-related adverse reactions. Previous studies have shown that risperidone use is associated with a dose-dependent elevation of prolactin in SZ patients. For example, a study of young men treated with risperidone showed that 68% of patients with SZ had prolactin levels above the upper limit of normal and that the effect of risperidone on prolactin was dose-dependent [38]. In another study of adolescent patients with autism, prolactin levels were found to be lower in the low-dose risperidone group than in the high-dose risperidone group [39]. Our large study of 198 patients with FEMN showed that a low dose of risperidone in the RS group significantly alleviated the rapid increase in prolactin levels and reduced EPS symptoms compared to a high dose in the control group.

This study also demonstrated that risperidone combined with sertraline significantly improved psychosocial functioning in FEMN patients with SZ. In addition, improvements in psychotic symptoms, depressive symptoms, and changes in serum prolactin levels were associated with improvements in psychosocial functioning. Patients with SZ usually show deficits in various psychosocial domains, such as the quality of social relationships, self-care abilities, and occupational domains [40–43]. As a result, they are at risk of unemployment, less likely to engage in social activities, and usually have no or few satisfying intimate relationships [44]. Our study is consistent with previous studies, showing that improvements in psychosocial functioning are associated with improvements in clinical symptoms and adverse reactions in SZ patients [45, 46]. Notably, a previous study by our group showed that low-dose ziprasidone combined with sertraline was efficacious for psychotic symptoms and psychosocial functioning in FEMN SZ, with fewer adverse effects compared to standardized doses of ziprasidone [47], suggesting that the combination therapy approach is also applicable to other types of antipsychotics and that the use of a low-dose antipsychotics in combination is a better option in patients with SZ.

In the present study, we noted some limitations. The first limitation was that this was not completely a double-blind placebo-controlled study. Although the nurses did not tell the patients what medications they were taking and the patients did not mutually know the number of capsules used, the patients may have identified the intervention because the RS group took two capsules and the control group took one capsule. Second, a study design comprises 4 groups, including regular dose risperidone monotherapy, low-dose risperidone monotherapy, regular dose risperidone plus sertraline, and low-dose risperidone plus sertraline will be better to answer if sertraline can have additional benefits, either with a better effect on improved psychotic/affective symptoms or lower adverse effects. Third, this study was a 24 week clinical trial, which is too short to draw firm conclusions. In particular, in this study, sertraline was administrated during psychotic onset and not throughout the whole treatment of SZ, which is a chronic disorder that usually requires long-term treatment with antipsychotics. Therefore, since the investigation was conducted solely during the first 24 weeks of treatment, there should not be any generalizations beyond this period. Fourth, another limitation was to extrapolate the results obtained on every other antipsychotic medication, as they can significantly differ in their mechanisms of action. Fifth, the study population was limited to Asians, which may limit the generalization of the findings in this study to other populations.

In conclusion, the present study suggests that low-dose risperidone combined with sertraline has a better therapeutic effect on psychotic symptoms and social functioning in FEMN SZ patients than standard doses of risperidone alone. Our study is the first to report that sertraline combined with low-dose risperidone is superior to conventional therapeutic doses of risperidone, providing a new and clinically meaningful contribution to the treatment of the early stages of SZ. These findings are promising, however, further multicenter studies of antipsychotic drug combinations in patients with FEMN SZ are warranted to confirm the preliminary findings.

## Supplementary Information

**Additional file 1: Figure 1.** Flow diagram of included studies.

## Data Availability

The datasets generated and analyzed during the current study are available from the corresponding author upon reasonable request.

## Abbreviations

SZ: Schizophrenia
FEMN: First-episode and medication-naïve
RS group: Low-dose risperidone in combination with sertraline
PANSS: Positive and negative syndrome scale
HAMD: Hamilton depression rating scale
PSP: Personal and social performance scale
CATIE: The national institute of mental health initiated the clinical antipsychotic trials of intervention effectiveness
SSRI: Selective 5-hydroxytryptamine reuptake inhibitor
5-HT: Serotonin
DSM-IV: Diagnostic and statistical manual of mental disorders-IV
ECG: Electrocardiogram
ESRS: Extrapyramidal symptom rating scale
ANOVA: One-way analysis of variance
RM-MANOVA: Repeated measures multivariate analysis of variance
