# Supplementary figures and images for: Efficacy of low-dose risperidone in combination with sertraline in first-episode drug-naïve patients with schizophrenia: a randomized controlled open-label study
Source: J Transl Med. 2023 Jul 4;21:432. doi: 10.1186/s12967-023-04272-7 (PMC10318661; doi:10.1186/s12967-023-04272-7)

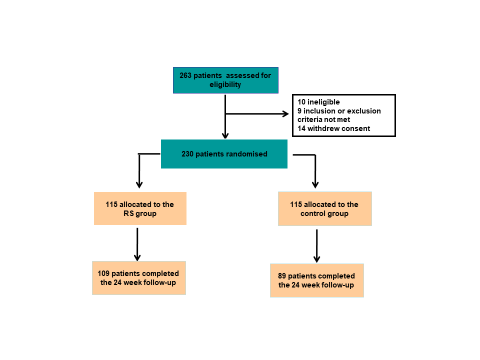

Supplement: Supplementary file 1 — Additional file 1: Figure 1. Flow diagram of included studies. [file 12967_2023_4272_MOESM1_ESM.docx]
